# Combined Transplantation of Olfactory Ensheathing Cells With Rat Neural Stem Cells Enhanced the Therapeutic Effect in the Retina of RCS Rats

**DOI:** 10.3389/fncel.2020.00052

**Published:** 2020-03-24

**Authors:** Wei Zhai, Lixiong Gao, Linghui Qu, Yijian Li, Yuxiao Zeng, Qiyou Li, Haiwei Xu, Zheng Qin Yin

**Affiliations:** ^1^Southwest Hospital/Southwest Eye Hospital, Third Military Medical University (Army Medical University), Chongqing, China; ^2^Key Lab of Visual Damage and Regeneration & Restoration of Chongqing, Chongqing, China; ^3^Department of Ophthalmology, The 6th Medical Center of PLA General Hospital, Beijing, China

**Keywords:** retinal degenerative diseases, neural stem cells, olfactory ensheathing cells, combined transplantation, RCS rats

## Abstract

Retinal degenerative diseases (RDDs) are the leading causes of blindness and currently lack effective treatment. Cytotherapy has become a promising strategy for RDDs. The transplantation of olfactory ensheathing cells (OECs) or neural stem cells (NSCs) has recently been applied for the experimental treatment of RDDs. However, the long-term outcomes of single-cell transplantation are poor. The combined transplantation of multiple types of cells might achieve better effects. In the present study, OECs [containing olfactory nerve fibroblasts (ONFs)] and NSCs were cotransplanted into the subretinal space of Royal College of Surgeons (RCS) rats. Using electroretinogram (ERG), immunofluorescence, Western blot, and *in vitro* Transwell system, the differences in the electrophysiological and morphological changes of single and combined transplantation as well as the underlying mechanisms were explored at 4, 8, and 12 weeks postoperation. In addition, using the Transwell system, the influence of OECs on the stemness of NSCs was discovered. Results showed that, compared to the single transplantation of OECs or NSCs, the combined transplantation of OECs and NSCs produced greater improvements in b-wave amplitudes in ERGs and the thickness of the outer nuclear layer at all three time points. More endogenous stem cells were found within the retina after combined transplantation. Glial fibrillary acidic protein (GFAP) expression decreased significantly when NSCs were cotransplanted with OECs. Both the vertical and horizontal migration of grafted cells were enhanced in the combined transplantation group. Meanwhile, the stemness of NSCs was also better maintained after coculture with OECs. Taken together, the results suggested that the combined transplantation of NSCs and OECs enhanced the improvement in retinal protection in RCS rats, providing a new strategy to treat RDDs in the future.

## Introduction

Characterized by the progressive loss or malfunction of retinal cells, retinal degenerative diseases (RDDs) are the leading causes of blindness. One of the most common RDDs is the age-related macular degeneration (AMD) (Veleri et al., 2015). There are two types of AMD, the dry AMD and the wet AMD, whose pathological characteristics are the neovascularization secondary to the stenosis of choroidal vessels and the decrease in phagocytosing function of retina pigment epithelium (RPE) followed by photoreceptor death, respectively (Blasiak, 2020). Data have shown that the general prevalence of all types of AMD is ∼8.7%, and the number of individuals affected by AMD will increase to 196 million in 2020 (Wong et al., 2014; Jonas et al., 2017). Currently, there are no specific therapeutic methods for AMD, especially for dry AMD. Treatments for wet AMD such as intravitreous injection do not stop the degeneration of the retina in wet AMD patients (Mavija et al., 2014). As retinal cell loss is the ultimate result of AMD, stem cell therapy is becoming a promising method for treating AMD (Nazari et al., 2015). To better understand the diseases, several RDD models have been developed. Royal College of Surgeons (RCS) rat is one of them. Characterized by the inability of RPE to phagocytose photoreceptor outer segments, RCS rat is the first known animal with inherited retinal degeneration (Strauss et al., 1998). The mutation in this model is found to be the deletion in the receptor tyrosine kinase gene Mertk, which links to the phagocytosing function of RPE (D’Cruz et al., 2000). The RPE dysfunction will further lead to the deposition of photoreceptor outer segments and consequent photoreceptor degeneration. Since its pathology is similar to RDDs, RCS rat is widely used as an animal model to mimic AMD and retinitis pigmentosa (LaVail, 2001). The progress of retinal degeneration is rapid in RCS rats. The response of electroretinograms (ERGs) in RCS rat begin to decrease at postnatal 21 days, reduce by half at postnatal 50 days, and nearly disappear at postnatal 100 days (Pinilla et al., 2005).

As adult stem cells within the central nervous system (CNS), neural stem cells (NSCs) can generate both neurons and glia (Obernier and Alvarez-Buylla, 2019). Several studies have confirmed the therapeutic effect of NSCs after transplantation into the injured CNS (Marsh and Blurton-Jones, 2017). McGill et al. showed that the transplantation of NSCs into the subretinal space (SRS) of RCS rats preserves retinal function and protects photoreceptors from death through the phagocytosis of photoreceptor outer segments (McGill et al., 2012). Lin et al. (2014) found that NSCs have a better proliferative ability than that of retinal progenitor cells (RPCs). Moreover, upon treatment with transforming growth factor beta type III and retinoic acid, NSCs are able to differentiate into opsin-positive retinal cells (Lin et al., 2014). Our previous work also confirmed the preservative effect of the subretinal transplantation of NSCs in rd1 mice (Li et al., 2016). However, there were several problems associated with transplanting NSCs alone into the retina: evidence of functional improvement was only observed during a small treatment window, transplantation triggered gliosis, and the migration of grafted cells was very limited. The combined transplantation of two or more different types of cells might improve the therapeutic effect of stem cell transplantation. Our previous work showed that the combined transplantation of mesenchymal stem cells and RPCs can simultaneously generate synergistic effects after subretinal transplantation (Qu et al., 2017).

As a physiological response to lesion to the CNS, gliosis is a double-edged sword. In the retina, the gliotic response from Müller cells regulates the size of the glial scar, which inhibits transplanted cells from exerting their therapeutic effect. Our previous results showed that olfactory ensheathing cells (OECs), a glial cell type originating from the neocortex of the olfactory bulb, inhibits gliosis in the retinas of RCS rats (Xie et al., 2017). Therefore, transplanting OECs with NSCs might improve the restoration of visual function in RCS rats.

OECs have been reported to support the continuous growth and regeneration of olfactory axons throughout life (Ramon-Cueto and Avila, 1998). Robust studies have confirmed that transplanted OECs exhibit neuroplastic and neuroregenerative effects via interacting with the glial scar and stimulating angiogenesis, axonal outgrowth, and remyelination in the spinal cord injury (Roet and Verhaagen, 2014; Gomez et al., 2018). OECs have also been found to protect visual function. Our previous work showed that OECs restore retinal function and alleviate retinal degeneration in RDD animal models via reducing the gliotic injury response of Müller cells, phagocytosing retinal outer segments, and inhibiting oxidative stress (Huo et al., 2011, 2012; Xie et al., 2017; Xue et al., 2017). We further confirmed that OECs can promote retinal ganglion cell survival and axonal regeneration after optic nerve injury for 3 months (Wang et al., 2017). However, the protective effect of OECs in the eyes is maintained for a relatively short period (Xue et al., 2017). The capacity of OECs to migrate relies on various factors (Gomez et al., 2018). These limitations restrict research on of the treatment of RDDs with OECs.

Because of the potential improvement in the gliotic microenvironment of OECs as well as the therapeutic effect of NSCs, we hypothesized that the cotransplantation of these two cell types might produce a better effect. In the present study, NSCs and OECs were transplanted either singly or in combination into the SRS of RCS rats at early degenerative stage. Using ERGs, immunofluorescence, Western blotting, and an *in vitro* Transwell system, we discovered the efficacy of combined transplantation and explored the possible underlying mechanisms at 4, 8, and 12 weeks postoperation. These three time points covered the moderate to the severe retinal degeneration of RCS rats.

## Materials and Methods

### Animals and Ethics

The RCS (28 days) and Long Evans (LE) rats were obtained from the Animal Research Center of the Third Military Medical University (TMMU). Rats were raised under a 12-h light/dark cycle in the specific pathogen-free room of the Animal Care Center of the First Affiliated Hospital of TMMU. The breeding of LE rats was performed to harvest embryos as well as the neonatal LE rats. All tissue collection and experimental procedures were performed according to protocols approved by the Institutional Review Board of the TMMU and conformed to the National Institutes of Health (NIH) guidelines on the ethical use of animals.

### *N* Values and Blinding

For *in vivo* study, 18 animals underwent transplantation treatment in each transplantation group at the starting point. On each of the three different posttransplantation time points, six animals in each group were killed after recording ERG. Three animals were used for immunofluorescent test and three animals for Western blot test. In summary, the *N* value in ERG test was 6; in immunofluorescence, 3; and in Western blot, 3. For *in vitro* study, both immigration and differentiation tests were repeated three times (*N* = 3). Cell harvest was repeated three times in both cells, and identification of cells in each batch was performed to ensure their characteristics (*N* = 3). As for the randomization and blinding, all treatments were randomized, and the persons performing the transplantation surgeries and histological analysis were blinded with respect to the treatment condition.

### Isolation, Culture, and Identification of OECs

After LE rats (90 days old) were anesthetized with pentobarbital sodium (10 mg/kg, Sigma-Aldrich), the olfactory bulbs were dissected and removed under a microscope. The glomerular layers of the olfactory bulbs were carefully isolated and cut into small pieces. The tissues were digested in 0.1% trypsin for 15 min at 37°C, and the reaction was stopped by OEC culture medium containing Dulbecco’s modified Eagle’s medium/F-12 culture medium (DMEM/F-12, 1:1 mixture, HyClone) supplemented with 10% fetal bovine serum (FBS, Gibco) and a mixture of penicillin and streptomycin (PS, 1%, Gibco). Then, the OEC suspension was centrifuged at 1,500 rpm for 5 min and resuspended in OEC culture medium. Then, OECs were plated on 35-mm dishes coated with 10 μg/ml laminin and incubated in a 5% CO_2_ saturation-humidity atmosphere at 37°C. The culture medium was changed every 3 days. Subculturing was performed once the cell density was over 80%. OECs were identified at passage 3. After being digested by trypsin, plated on laminin-coated coverslips, and cultured for 3 days, OECs were identified via immunofluorescence. The details are described in section “Immunofluorescence.”

### Isolation, Culture, and Identification of NSCs

Neural stem cells were harvested from the visual cortex of embryonic LE rats at embryonic day 13.5 and cultured. The maternal LE rats were anesthetized with pentobarbital sodium (10 mg/kg, Sigma-Aldrich), and uteruses containing fetal rats were isolated. The visual cortexes of fetal rats were carefully dissected and cut into small pieces under a microscope. After being digested with Accutase (Innovative Cell Technologies, United States) for 5 min at 37°C and stopped by the NSC culture medium containing DMEM/F-12 (Hyclone) supplemented with B27 (Gibco), glutamine (Gibco), basic fibroblast growth factor (bFGF) (20 ng/ml, Peprotech), epidermal growth factor (EGF) (10 ng/ml, Peprotech), and a mixture of PS (1%, Gibco), NSC suspension was centrifuged at 4,000 rpm for 5 min and resuspended with the NSC culture medium. Then, NSC suspension was transferred to floating culture flasks and incubated in a 5% CO_2_ saturation-humidity atmosphere at 37°C. The culture medium was changed every 3 days. For identification, passage 3 floating spheres were directly plated on laminin-coated coverslips or plated after being digested into single cells and cultured for 3 days, after which NSCs were identified via immunofluorescence. The details are described in section “Immunofluorescence.”

For flow cytometry, passage 3 floating spheres were digested into single cells. After perforation, washing, and blocking, NSCs were divided into blank and experimental groups. The latter group was incubated with Nestin-FITC, Pax6-FITC, and Sox2-FITC primary antibodies. Then, all groups were tested by flow cytometry (BD, United States).

### *In vitro* Migration and Differentiation Assay of NSCs

Passage 3 NSCs were used to conduct both migration and differentiation assays. For the migration assay, Transwell systems and six-well plates were used. In detail, 2 × 10^5^ rhodamine (Sigma-Aldrich)-labeled NSCs alone or 2 × 10^5^ rhodamine-labeled NSCs together with 2 × 10^5^ Hoechst (Beyotime)-labeled OECs (NSCs + OECs) were added to a single Transwell system. The retinas of neonatal LE rats (postnatal day 0) were removed and isolated on ice after anesthetization with pentobarbital sodium (10 mg/kg, Sigma-Aldrich). The retinas were then placed onto the NSCs or NSCs + OECs in the Transwell system with 1 ml of NSC culture medium. After 12 days of coculture, the retinas were fixed with 4% paraformaldehyde (PFA) for 3 h, followed by dehydration with 30% sucrose for 12 h. Then, the retinas were embedded and sectioned into 10-μm slices. 4′,6-Diamidino-2-phenylindole (DAPI) staining was performed on retinal sections from the NSC group. The sections were observed under an immunofluorescence microscope. For the differentiation assay, Transwell systems and 24-well plates were used. In the control group, 1 × 10^5^ NSCs were plated along the Transwell membrane. In the experimental group, 2 × 10^5^ OECs were first plated in the plates, and 1 × 10^5^ NSCs were then plated on the upper Transwell membrane. All groups were cultured in the NSC culture medium and cultured for 24 h. In the bromodeoxyuridine (BrdU) test, 25 μl of BrdU solution (2 mg/ml) was added to the corresponding wells at 23 h. Then, Transwell membranes containing NSCs were examined via immunofluorescence. The details are described in section “Immunofluorescence.” These two assays were repeated three times.

### Cell Transplantation

Subretinal transplantation was performed as previously described (Qu et al., 2017). In brief, cell suspensions containing OECs, NSCs, or their combination were injected into the temporal subretinal space of the left eyes of RCS rats 4 weeks postnatally (5 μl/eye). The cell suspension in the combination group was a mixture containing 2.5 × 10^5^ cell NSCs and 2.5 × 10^5^ cells OECs/olfactory nerve fibroblasts (ONFs) per eye. In the single transplantation groups, 2.5 × 10^5^ cells of NSCs per eye were transplanted in the NSC group, and 2.5 × 10^5^ cells OECs/ONFs per eye were transplanted in the OEC group. An identical volume of 0.01 M phosphate-buffered saline (PBS) (5 μl) was injected into the temporal subretinal space of the right eyes of RCS rats. All transplanted cells were labeled with the fluorescent marker CM-DiI (2 mg/ml, Invitrogen). The pupils were dilated with 1% tropicamide (Santen Pharmaceutical Co., Ltd. Osaka, Japan) 30 min before surgery. Once the RCS rats were anesthetized (120 mg/kg ketamine and 20 mg/kg xylazine), a 10-μl Hamilton syringe (30 gauge; Hamilton, NV, United States) containing a cell suspension was tangentially inserted into the subretinal space through the conjunctiva and sclera, which led to a self-sealing wound tunnel. Paracentesis of the anterior chamber was performed to reduce the intraocular pressure and limit the efflux of cells from the injection site. Fundus examinations were performed immediately after the operations. The eyes that did not receive any treatment were labeled as the “blank” group. The eyes in which OECs, NSCs, or OECs + NSCs were transplanted were labeled as the “OEC group,” “NSC group,” or “OEC + NSC group,” respectively. The eyes that were injected with an equal amount of PBS were labeled as the “PBS” group.

### ERG Recording

ERG recording was performed 4, 8, and 12 weeks postoperation to evaluate the retinal functional changes, as previously described (Lin et al., 2014). In brief, after being dark adapted for at least 12 h, RCS rats were anesthetized by an intraperitoneal injection of a solution of ketamine (120 mg/kg) and xylazine (20 mg/kg). Pupils were dilated with 1% tropicamide before testing. A heating pad was used to maintain the body temperature at 37°C. Two active gold electrodes were placed on each cornea as recording electrodes. The reference and ground electrodes were subcutaneously inserted into the mid-frontal area of the head and tail, respectively. Light stimulations were delivered with a xenon lamp at 3.0 cd s/m^2^. The b-wave amplitudes were recorded and processed by a RETI-Port device (Roland Consult, Brandenburg, Germany). All procedures were performed in a dark room with dim red safety light. When dealing with the results, a and b waves were marked by the typical type of ERG waves as well as the latent period, which was basically achieved by computer and checked by experimenters. If the computer failed to calculate the proper point, experimenters would manually measure the results. Since b wave represents the transduction of extracellular currents and is considered to be the major component of the human ERG recording as used in clinical and experimental analysis of retinal function (Perlman, 1995), we typically focus on the amplitude of b wave in the present study.

### Morphological Preparation of Retina

After being anesthetized by 1% pentobarbital (150 mg/kg), RCS rats were perfused with normal saline and 4% PFA via the circulation system on 4, 8, and 12 postoperation weeks as we previously described (Qu et al., 2017). After being enucleated and fixed in 4% PFA for 3 h, eyeballs were incubated in 30% glucose solution overnight. During the embedding of the eyeball, we marked the injection site and placed the embedded eyeball on the slicer. This placement was carried out in two disciplines: (1) The eyeball stood vertically in the embedding medium; (2) the plane formed by 3 points (the injection site, optic disk, and the point opposite to the injection site on the eyeball) kept parallel to the blade (Supplementary Figure 1). These disciplines can ensure the consistency in the sections among different groups. Then, 10-μm serially frozen sections were carefully made.

### Immunofluorescence

Immunofluorescence of the identification of OECs/ONFs and NSCs as well as NSC differentiation assay were performed as previously described. In detail, after being rinsed in 0.01 M PBS, blocked in 10% of goat serum, and perforated in 0.1% of Triton X-100, coverlids and Transwell membrane were incubated with primary antibodies overnight at 4°C. The coverlids with OECs/ONFs were incubated with anti-P75 (1:500, mouse, Santa) antibodies; the coverlids with NSCs with anti-Nestin (1:500, rabbit, Abcam), anti-GFAP (1:500, mouse, Abcam), and anti-Tuj1 (1:1000, mouse, Beyotime) antibodies; and the Transwell membrane with NSCs with anti-GFAP (1:500, mouse, Abcam), anti-Sox2 (1:500, rabbit, Abcam), anti-Pax6 (1:500, rabbit, Santa), and anti-BrdU antibodies (1:500, Cell Signaling). Cy3-or 488-conjugated secondary antibodies, (Invitrogen) were then implemented (1:400, 37°C, 2 h). Before examination with a confocal laser scanning microscope (Leica, Germany), cells were counterstained with DAPI (Sigma Aldrich). Immunofluorescence of retina sections was also performed as previously described (Gao et al., 2015). In detail, after being washed in 0.01 M PBS, blocked in 10% of goat serum, and perforated in 0.1% of Triton X-100, selected sections were incubated with the primary antibodies, anti-GFAP (1:500, mouse, Abcam), anti-Sox2 (1:500, rabbit, Abcam), and anti-recoverin (1:1000, rabbit, Millipore) antibodies in 1% bovine serum albumin (BSA) at 4°C overnight. Cy3- or 488-conjugated secondary antibodies (Invitrogen) were then implemented (1:400, 37°C, 2 h). Before examination with a confocal laser scanning microscope (Leica, Germany), sections were counterstained with DAPI (Sigma Aldrich).

### Western Blot

Animals were euthanized with CO_2_ at 4, 8, and 12 weeks postoperation, after which eyeballs were enucleated and retinas were quickly isolated on ice. After being rinsed in 0.01 M PBS and drained, retina tissues were lysed in ice-cold tissue lysis buffer [10% phenylmethylsulfonyl fluoride (PMSF) + 90% radioimmunoprecipitation assay (RIPA)]. The lysates were then centrifuged at 15,000 rpm for 10 min at 4°C. Protein concentration was determined using the BCA Protein Assay (Beyotime). After boiling in loading buffer for 10 min, total proteins (10 μg per slot) were electrophoresed on a 12% sodium dodecyl sulfate polyacrylamide gel and then transferred onto polyvinylidene fluoride membranes. After being blocked in 5% fat-free milk for 2 h at 37°C, membranes were incubated with anti-GFAP antibody (1:500, rabbit, Abcam) and anti-glyceraldehyde 3-phosphate dehydrogenase (anti-GAPDH) (1:1,000, mouse, Proteintech Group) antibody overnight at 4°C. Membranes were then incubated with peroxidase-conjugated immunoglobulin G (1:2,000; Santa Cruz Biotechnology). After being washed in Tris-buffered saline with Tween-20 (TBS-T) and developed in developing solution, membranes were scanned using the Bio-Rad exploding system (Bio-Rad, CA, United States) with corresponding software.

### Outer Nuclear Layer Thickness Analysis

Six sections that were cut using the same horizontal angle across the optic disk were chosen to measure the thickness of the outer nuclear layer (ONL). From each section, an average of three areas of the temporal retina area of the optic disk was selected (Supplementary Figure 2). The thickness of the ONL was measured by ImageJ (NIH, United States). The average ONL thickness in the three areas represented the ONL thickness of the section.

### Quantitative Histological Analysis

To quantitatively analyze the differences in the expression of NSC markers after coculture with OECs, three comparable visual fields from each cell slide were randomly selected. The numbers of BrdU-, Pax6-, Sox2-, and GFAP-positive cells were manually counted and averaged. To quantitatively analyze *in vivo* cell migration after cell transplantation, six sections that were cut using the same horizontal angle across the optic disk were chosen to conduct the migration measurement. In each section, the photos of retina were taken under a 400× microscope. Integrate image of the whole retina was generated by splicing these photos. The distance that transplanted cells migrated within the SRS was measured by ImageJ (NIH, United States) in each integrate image. To quantitatively analyze the status of endogenous stem cell formation after transplantation, at least three sections across the optic disk were selected from each group after Sox2 staining. The number of Sox2-positive cells within a 150 μm × 150 μm square visual field was manually counted and averaged. To semiquantitatively analyze the GFAP expression level, at least three sections across the optic disk were selected from each group after GFAP staining. The density of GFAP-positive cells was recorded by ImageJ (NIH).

### Statistical Analysis

Using Statistical Product and Service Solutions software V17.0 (SPSS, Chicago, IL, United States), all quantitative results were analyzed by one-way ANOVA followed by Fisher’s protected least-significant difference *post hoc* tests. The data are presented as the mean ± standard error. *P* < 0.05 was considered statistically significant.

## Results

### Identification of Primarily Isolated OECs and NSCs

The identification of both OECs and NSCs was conducted before transplantation. The OECs and NSC were tested at passage 3. The nerve growth factor receptor P75 and Nestin were used as OECs and NSCs markers, respectively (Xie et al., 2017). The OECs were fusiform shaped with elongated processes (Supplementary Figures 3A,B). By immunofluorescence, we found that nearly half of the OEC/ONF mixture expressed P75, and the other half expressed FN (Supplementary Figures 3C–E). For NSCs, the results showed that, when floating culture was performed, NSCs exhibited a spherical shape and expressed Nestin (Supplementary Figures 4A,B). After being cultured in serum-free media for 2 weeks, NSCs differentiated into neurons and expressed Tuj1 (Supplementary Figure 4C). Few GFAP-positive glial cells were observed (Supplementary Figure 4D). Flow cytometry showed that a high percentage of NSCs expressed Sox2 (98.86%), Pax6 (98.94%), and Nestin (98.38%) (Supplementary Figure 4E). These results confirmed the characteristics of harvested OECs/ONFs and NSCs.

### Combined Transplantation Enhanced Electrophysiological Improvement of RCS Rats

**FIGURE 1:**
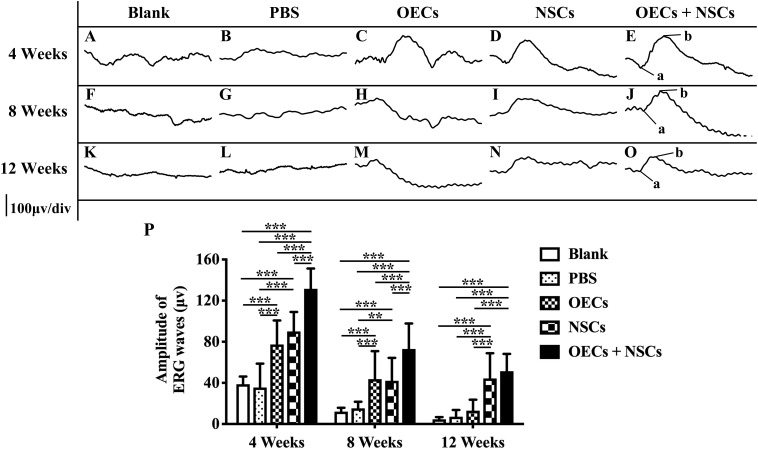
Electrophysiological improvement after combined transplantation of rat olfactory ensheathing cells (OECs) and neural stem cells (NSCs) into the subretinal space of Royal College of Surgeons (RCS) rats. **(A–E)** Representative electroretinogram (ERG) results in the blank, PBS, OEC, NSC, and OEC + NSC groups at 4 weeks postoperation. **(F–J)** Representative ERG results in the blank, PBS, OEC, NSC, and OEC + NSC groups at 8 weeks postoperation. **(K–O)** Representative ERG results in the blank, PBS, OEC, NSC, and OEC + NSC groups at 12 weeks postoperation. **(P)** Relative statistical analysis of all groups. a and b point in **(E,J,O)** indicated the a and b waves, which represented the light absorption of photoreceptors and postsynaptic responses of photoreceptors, respectively. ^∗∗^*P* < 0.01; ^∗∗∗^*P* < 0.001.

After transplantation with a single cell type or a combination of the two cell types, ERGs were recorded, and b-wave amplitude was measured to determine electrophysiological improvements at 4, 8, and 12 weeks postoperation. The results showed that, compared to the blank and the PBS group, the OEC, NSC, and OEC + NSC groups presented significant improvements in b-wave amplitudes at all time points (*P* < 0.001; Figures 1A–P). Moreover, there were also differences among three transplantation groups. In particular, at 4 weeks postoperation, the OEC + NSC group showed significantly higher b-wave amplitude than that in either the OEC or the NSC group (*P* < 0.001, *P* < 0.001; Figures 1C–E,P). There was no significant difference between the OEC and the NSC group (*P* > 0.05, Figures 1C–E,P). At 8 weeks postoperation, the OEC + NSC group still presented significantly higher b-wave amplitude than that in either the OEC or the NSC group (*P* < 0.05, *P* < 0.05; Figures 1H–J,P). There was no significant difference between the OEC and the NSC group at 8 weeks postoperation (*P* > 0.05; Figures 1H–J,P). However, the retina-function restoration became different at 12 weeks postoperation. Compared to the OEC group, the OEC + NSC group showed a significant increase in b-wave amplitude (*P* < 0.001; Figures 1M–O,P), while no statistically significant difference was observed between the NSC and the OEC + NSC group (*P* > 0.05; Figures 1N–P). In addition, the NSC group also presented significantly higher b-wave amplitude than that of the OEC group at 12 weeks postoperation (*P* > 0.05; Figures 1M,N,P). These results indicated that, compared with the single transplantation, the combined transplantation of OECs and NSCs enhanced the electrophysiological improvement of RCS.

### Combined Transplantation Showed Better Photoreceptor Protection in RCS Rats

**FIGURE 2:**
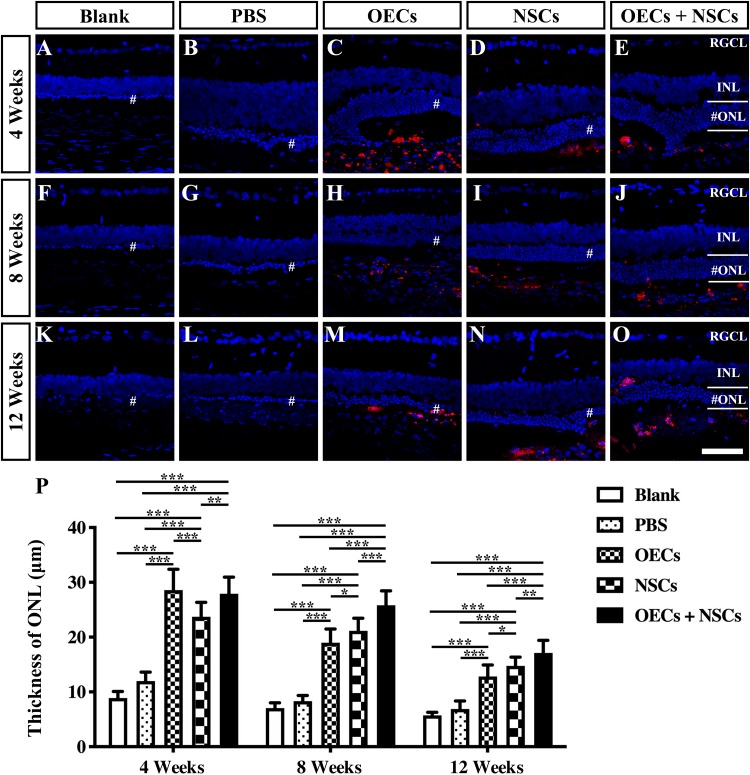
Protection of ONL after combined transplantation of rat olfactory ensheathing cells (OECs) and neural stem cells (NSCs) into the subretinal space of Royal College of Surgeons (RCS) rats. **(A–E)** Retina sections with 4′,6-diamidino-2-phenylindole (DAPI) staining in the blank, PBS, OEC, NSC, and OEC + NSC groups at 4 weeks postoperation. **(F–J)** Retina sections with DAPI staining in the blank, PBS, OEC, NSC, and OEC + NSC groups at 8 weeks postoperation. **(K–O)** Retina sections with DAPI staining in the blank, PBS, OEC, NSC, and OEC + NSC groups at 12 weeks postoperation. The mark “#” represented the out nuclear layer. **(P)** Statistical analysis of the ONL thickness in transplantation groups. Scale bar: **(A–O)** 50 μm. ^∗^*P* < 0.05; ^∗∗^*P* < 0.01; ^∗∗∗^*P* < 0.001.

**FIGURE 3:**
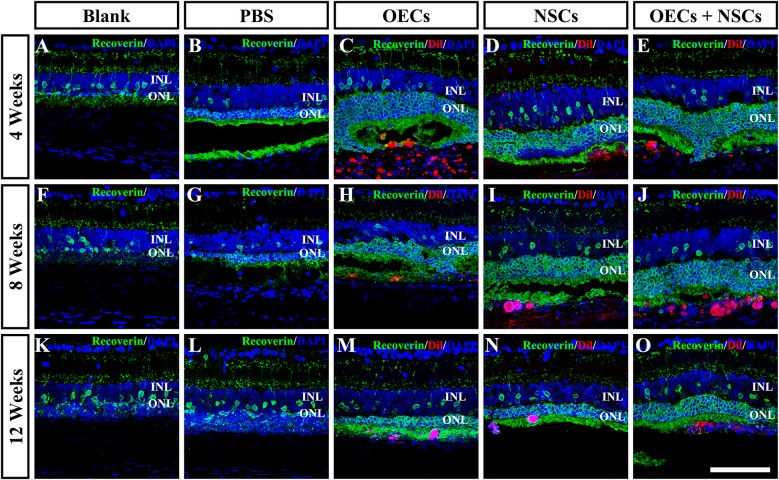
Protection of photoreceptors after combined transplantation of rat olfactory ensheathing cells (OECs) and neural stem cells (NSCs) into the subretinal space of Royal College of Surgeons (RCS) rats. **(A–E)** Immunofluorescence of Recoverin in the blank, PBS, OEC, NSC, and OEC + NSC groups at 4 weeks postoperation. **(F–J)** Immunofluorescence of Recoverin in the blank, PBS, OEC, NSC, and OEC + NSC groups at 8 weeks postoperation. **(K–O)** Immunofluorescence of Recoverin in the blank, PBS, OEC, NSC, and OEC + NSC groups at 12 weeks postoperation. Scale bar: **(A–O)** 75 μm.

To further evaluate the morphological effects of cell transplantation, the ONL thickness was measured. Compared with the blank and the PBS group, three transplantation groups all presented significant protective effects on the ONL (*P* < 0.001; Figures 2A–P). Among the transplantation groups, both the OEC + NSC and the OEC groups showed significantly better protective effects on the ONL than that of the NSC group at 4 weeks postoperation (*P* < 0.01, *P* < 0.05; Figures 2C–E,P). No significant difference was found between the OEC + NSC and the OEC group at this time point (*P* > 0.05; Figures 2C,E,P). At 8 weeks postoperation, the OEC + NSC group displayed a significant increase in ONL thickness compared to that of either the OEC or the NSC group (*P* < 0.001, *P* < 0.001; Figures 2H–J,P). There was no significant difference between the OEC and the NSC group at this time point (*P* > 0.05; Figures 2H,I,P). A similar change was found at 12 weeks postoperation. The OEC + NSC group still presented a significant higher ONL thickness than that of the two single transplantation groups (OECs, *P* < 0.001; NSCs, *P* < 0.05; Figures 2M–O,P). No significant difference was found between the two single transplantation groups (*P* > 0.05; Figures 2M–O,P). Moreover, when retinal sections were stained with recoverin to identify photoreceptors, all three cell transplant groups showed evidence of increased numbers of photoreceptors at each time point examined (Figures 3A–O). Taken together, these results suggested that combined transplantation showed a better protective effect on the ONL than single transplantation.

### Endogenous Stem Cell Activation After Combined Transplantation

**FIGURE 4:**
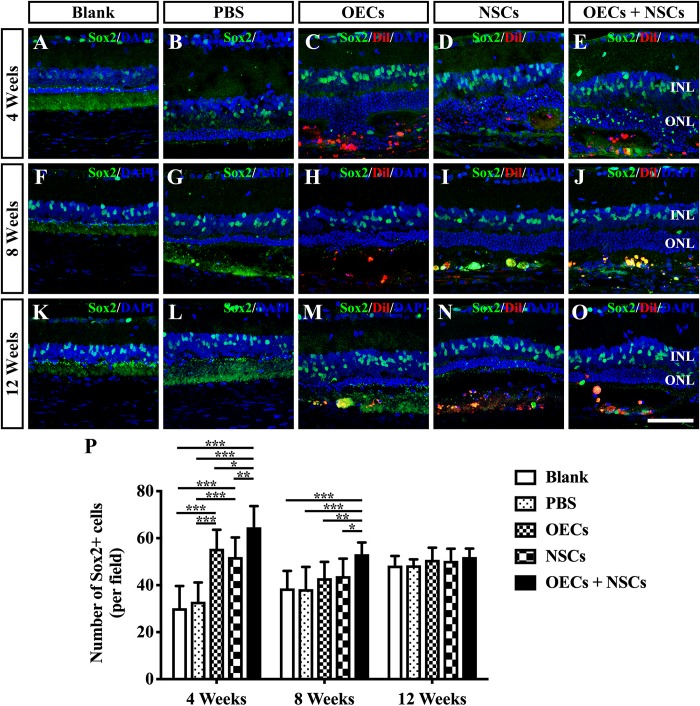
Activation of endogenous retinal stem cells after combined transplantation of rat olfactory ensheathing cells (OECs) and neural stem cells (NSCs) into the subretinal space of Royal College of Surgeons (RCS) rats. **(A–E)** Immunofluorescence of Sox2 in the blank, PBS, OEC, NSC, and OEC + NSC groups at 4 weeks postoperation. **(F–J)** Immunofluorescence of Sox2 in the blank, PBS, OEC, NSC, and OEC + NSC groups at 8 weeks postoperation. **(K–O)** Immunofluorescence of Sox2 in the blank, PBS, OEC, NSC, and OEC + NSC groups at 12 weeks postoperation. **(P)** Statistical analysis of the numbers of Sox2-positive cells in transplantation groups. Scale bar: **(A–O)** 50 μm. ^∗^*P* < 0.05; ^∗∗^*P* < 0.01; ^∗∗∗^*P* < 0.001.

To investigate the possibility that endogenous stem cell responses might underlie the apparent therapeutic actions of cell transplantation, we labeled retinas with Sox2 antibodies (Tian et al., 2011). The results showed that Sox2-positive cells within the retina were evenly distributed in the inner nuclear layer. At 4 weeks post-operation, three transplantation groups all presented a significant higher number of Sox2-positive cells than that of either the Blank group or the PBS group (*P* < 0.001; Figures 4A–E,P). Among the three transplantation groups, the number of Sox2-positive cells in the OEC + NSC group was significantly higher than that of either the OEC group or the NSC group at 4 weeks post-operation (*P* < 0.05, *P* < 0.01; Figures 4C–E,P). This significant difference lasted until 8 weeks post-operation (*P* < 0.01, *P* < 0.01; Figures 4H–J,P), while no significant differences in Sox2 expression were observed among the Blank group, the PBS, and the two single transplantation groups at this time point (*P* < 0.05; Figures 4F–J,P). However, by 12 weeks post-operation, there was no significant difference among groups (*P* < 0.05; Figures 4K–O,P). These results suggested that combined transplantation activated more endogenous stem cells at the early stage of transplantation.

### Influence on the Reactive Gliosis of Müller Cells After Combined Transplantation

**FIGURE 5:**
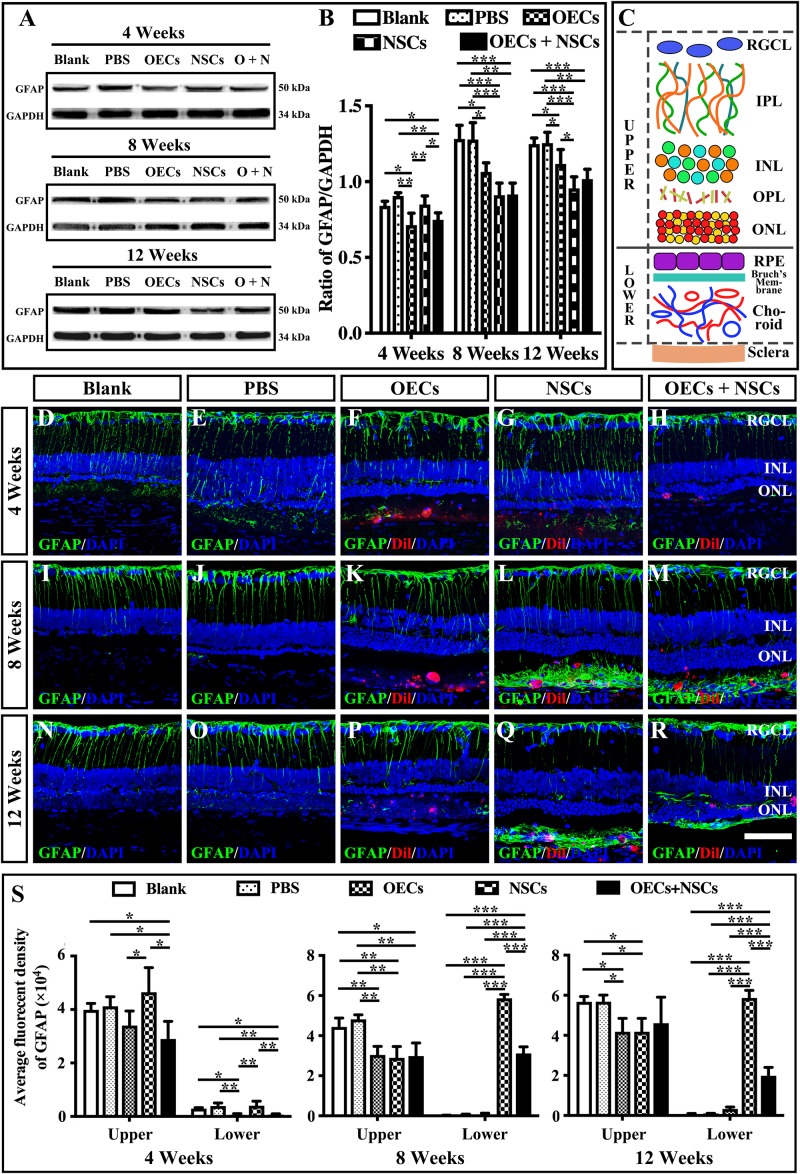
Influence of the reactive gliosis of Muller cells after combined transplantation of rat olfactory ensheathing cells (OECs) and neural stem cells (NSCs) into the subretinal space of Royal College of Surgeons (RCS) rats. **(A,B)** The Western blot analysis of glial fibrillary acidic protein (GFAP) expression after transplantation. **(A)** The band of GFAP and glyceraldehyde 3-phosphate dehydrogenase (GAPDH) at 4, 8, and 12 weeks postoperation. **(B)** Corresponding analysis of the Western blot results. **(C)** Schematic diagram of the division of the retina. Solid line indicated the boundary of the division. **(D–H)** Immunofluorescence of GFAP in the blank, PBS, OEC, NSC, and OEC + NSC groups at 4 weeks postoperation. **(I–M)** Immunofluorescence of GFAP in the blank, PBS, OEC, NSC, and OEC + NSC groups at 8 weeks postoperation. **(N–R)** Immunofluorescence of GFAP in the blank, PBS, OEC, NSC, and OEC + NSC groups at 12 weeks postoperation. **(S)** The average fluorescent intensity in the blank, PBS, OEC, NSC, and OEC + NSC groups of the upper and lower parts of the retina. RGCL, retinal ganglion cell layer; IPL, inner plexiform layer; INL, inner nuclear layer; OPL, outer plexiform layer; ONL, outer nuclear layer. Scale bar: **(A–O)** 50 μm. ^∗^*P* < 0.05; ^∗∗^*P* < 0.01; ^∗∗∗^*P* < 0.001.

The reactive gliosis of Müller cells following retina damage become an obstacle to retinal regeneration. To generally analyze the gliosis of Müller cells, we performed the Western blot analysis and the immunofluorescence of GFAP at 4, 8, and 12 weeks posttransplantation. WB results showed that at 4 weeks postoperation, the blank, the PBS, and the NSC groups presented similar GFAP expression levels, which were significantly higher than those in the OEC group and the OEC + NSC group (*P* < 0.05, *P* < 0.01; Figures 5A,B). However, at 8 weeks postoperation, GFAP expression level in three transplantation groups all significantly decreased compared to that in the blank and the PBS groups (OEC, *P* < 0.05; NSC, *P* < 0.001; OEC + NSC, *P* < 0.001; Figures 5A,B). The situation remained similar at 12 weeks postoperation; the only difference was that compared with the OEC group, the NSC group presented a significantly lower GFAP expression level (*P* < 0.05, Figures 5A,B).

We also divided the retina into upper and lower parts when analyzing the results of immunofluorescence (Figure 5C). The boundary was set to be the outer border of ONL (the inner border of SRS) (solid line in Figure 5C). The fluorescence intensity (FI) within the inner part and the outer part of the retina was measured and labeled as upper and lower, respectively (Figure 5C). Results showed that the trends of GFAP FI in the upper part of the retina were similar to the trends of the GFAP expression level in WB at all three time points (Figures 5B,S). Interestingly, the results of GFAP FI in the lower part of the retina showed distinct differences. At 4 weeks postoperation, the OEC and the OEC + NSC group presented significantly lower GFAP FIs than those in the blank, the PBS, and the NSC groups, respectively (*P* < 0.05, *P* < 0.01, *P* < 0.01; Figures 5D–H,S). There was no significant difference among the blank, the PBS, and the NSC groups. However, when it came to 8 and 12 weeks postoperation, situation changed dramatically. The NSC and OEC + NSC groups both showed significant increases in GFAP FI compared to those in the blank, the PBS, and the OEC groups at these two time points (*P* < 0.001, *P* < 0.001, *P* < 0.001; Figures 5I–S). While the OEC + NSC group displayed a significant decrease in GFAP FI compared to that in the NSC group (*P* < 0.001; Figures 5L,M,Q,R,S). Taken together, transplantation of OECs and NSCs can decrease the gliosis following retinal degeneration. Moreover, transplantation of NSCs would bring gliosis to the SRS, which could be inhibited by cotransplantation of OECs.

### The Possible Rescuing Mechanism of the Combined Transplantation

**FIGURE 6:**
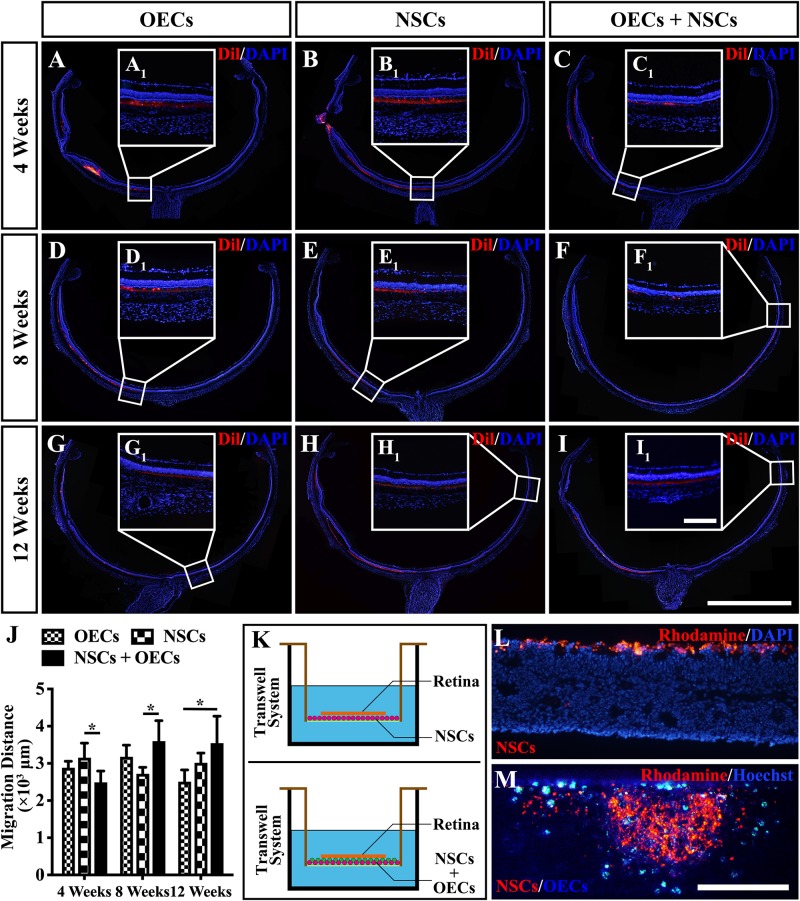
Migration of transplanted cells after combined transplantation of rat olfactory ensheathing cells (OECs) and neural stem cells (NSCs) into the subretinal space of Royal College of Surgeons (RCS) rats. **(A–C)** General microscopy of the retina section in the OEC, NSC, and OEC + NSC groups at 4 weeks postoperation. **(D–F)** General microscopy of the retina section in the OEC, NSC, and OEC + NSC groups at 8 weeks postoperation. **(G–I)** General microscopy of the retina section in OEC, NSC, and OEC + NSC groups at 12 weeks postoperation. **(A_1_–I_1_)** Relative partial enlargements showed the farthest location of transplanted cells. **(J)** Relative statistical analysis of transplantation groups. **(K)** The schematic diagram of the migration assay performed via Transwell system. **(L,M)** The results of migration analysis. NSCs were stained by Rhodamine, and OECs were stained by Hoechst. Cell migration with NSCs alone **(L)** as well as OECs and NSCs mixture **(M)** were tested. Scale bar: **(B,C)** 200 μm. Scale bar: **(A–I)** 2 mm; **(A_1_–I_1_)** 200 μm; **(L–M)** 200 μm.*P < 0.05.

**FIGURE 7:**
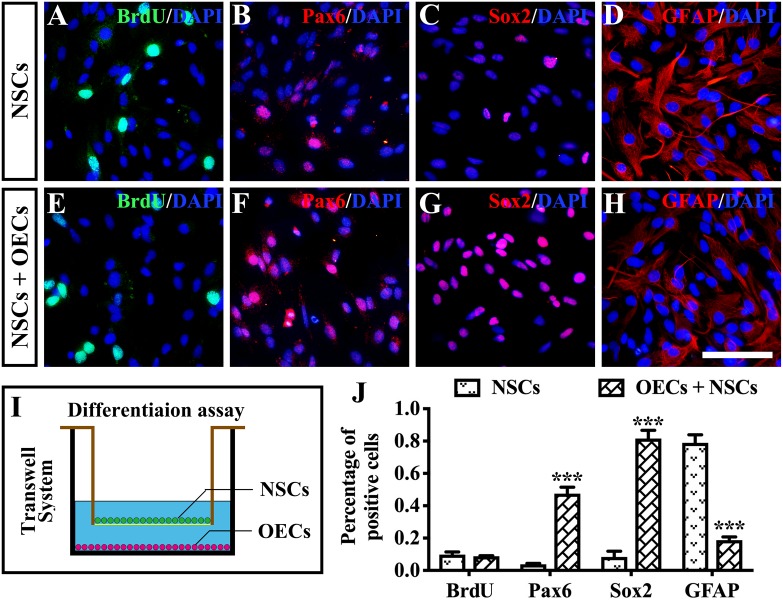
The influence on differentiation of neural stem cells (NSCs) after *in vitro* coculture with olfactory ensheathing cells (OECs) via Transwell system. **(A–H)** Immunofluorescence analysis of BrdU, Pax6, Sox2, and glial fibrillary acidic protein (GFAP) in NSCs cultured along group **(A–D)** and NSCs + OECs coculture group **(E–H)**. **(I)** The schematic diagram of the differentiation assay performed via Transwell system. **(J)** Relative statistical analysis of **(A–H)**. Scale bar: **(A–H)** 25 μm. ***P < 0.001.

To further investigate the mechanism of rescue following cotransplantation, we evaluated the influence of migration and the cell state of NSCs upon coculture with OECs. The migration of transplanted cells within the SRS was first investigated. The results showed that the OEC + NSC group presented a significantly shorter migration distance compared to that of the NSC group at 4 weeks postoperation (*P* < 0.05; Figures 6B,C,J). No significant difference was detected between the two single transplantation groups (*P* > 0.05; Figures 6A,B,J). However, by 8 and 12 weeks postoperation, the OEC + NSC group presented a significant increase in migration distance compared to that of the single transplantation groups (*P* < 0.05; Figures 6D–J). There was no significant difference between the two single transplantation groups at these two time points (*P* > 0.05; Figures 6D–H,J). An *in vitro* Transwell system was used to investigate the cell migration within the retina tissue (Figure 6K). After 12 days of culture, we found that there was little vertical migration of NSCs in the retinal tissue from the NSCs alone group (Figure 6L), while in the coculture group, there were many more cells that entered the retinal tissue (Figure 6M). Both *in vivo* and *in vitro* analyses confirmed the cell-migration-enhancement effect following cotransplantation. To further explore the influence of the cell state of NSCs following cotransplantation, coculture of OECs and NSCs via Transwell system was performed (Figure 7I). Results showed no significant difference in proliferation between the NSC single culture group and the NSC + OEC coculture group (*P* > 0.05; Figures 7A,E,J). However, the NSC + OEC coculture group presented significant higher Pax6 and Sox2 expressions than those of the NSC single culture group (Pax6, *P* < 0.001; Sox2, *P* < 0.001; Figures 7B,C,F,G,J). Meanwhile, a significant decrease in GFAP expression was also found in the NSC + OEC coculture group, compared to the NSC single culture group (*P* < 0.001; Figures 7D,H,J). These results suggested that NSCs exhibited enhanced stemness but reduced gliotic tendency when cocultured with OECs.

## Discussion

In the current study, the combined transplantation of OECs and NSCs produced a better neuroprotective effect and delayed retinal degeneration in a more effective way than single-cell transplantation in the retinas of RCS rats. Endogenous stem cell activation, enhanced migration of transplanted cells, and stemness maintenance of NSCs following combined transplantation may be possible underlying mechanisms. Comparing to the normal amplitude of ERG b wave in age-corresponding LE rats (about 1,300 ± 200 μV) (Xue et al., 2017), the ratios of the b-wave amplitude between the combined transplantation group and the normal LE rats at 4, 8, and 12 weeks postoperation were 10.1, 5.6, and 4%, respectively. Although positive influence was within a limited extent and dropped obviously with time, the better improvement reflected by ERG still lasted for 8 weeks, which is of great significance from bench to bedside. These results indicated that the combined transplantation of OECs and NSCs may be an alternative stem cell therapy for patients with RDDs in the future.

However, in the present study, the observation time lasted for only 12 weeks. The continued functional and morphological improvements were not observed. In addition, no direct measure of visual function was conducted in the present study, which could lead to incomplete assessment and functional bias. More importantly, limited observation period was not sufficient to discover underlying tumor formation possibility and other safety problems. In the future, studies with longer observation period and functional or behavioral tests need to be conducted.

Our previous research confirmed the visual restorative effect of OECs (Huo et al., 2012). The inhibition of gliosis via the downregulation of the Notch signaling pathway in Müller cells might be a possible mechanism (Xie et al., 2017). However, in this study, we found that combined transplantation of OECs and NSCs showed a better preservative effect than that of the OEC single transplantation, and this effect lasted for 8 weeks after transplantation.

Differences in the endogenous stem cell activation may be one of the explanations. In the spinal cord injury, the activation of endogenous stem cells was considered a promising method for spinal cord recovery (Qin et al., 2015). In the retina, Müller cells were reported to be reprogrammed as the progenitor cells and repair the degenerated retina (Jorstad et al., 2017; Yao et al., 2018). Under physiological conditions, Müller cells remain quiescent to avoid depletion of stem-cell pool. On the contrary, they are able to exit from latent state to proliferate and differentiate upon injuries (Madelaine and Mourrain, 2017). Our previous study found that the dedifferentiation of Müller cells was increased after the transplantation of retinal stem cells into the SRS of RCS rats, bringing both morphological and functional improvement to the host (Tian et al., 2011). Besides, Sox2 was identified to be re-expressed in Müller cells after injury, indicating that Müller cells exited the quiescent state and generated new retinal neurons (Gorsuch et al., 2017). As more Sox2-positive cells within the inner nuclear layer were found after combined transplantation in the present study, stronger endogenous repair of retina following activation of more endogenous stem cells might be an underlying mechanism.

Retinal gliosis is a non-neoplastic retinal glial proliferation followed by a complex retinal response participated by Müller cells, microglia cells, and alterations of the vasculature (Bringmann et al., 2006). It acts as a double-edged sword in the pathological process of RCS rats. On the one hand, reactive gliosis is a physical process that can be regarded as a cellular response to protect the retina from further damage. In addition, moderate gliosis can promote retinal repair following pathological insult (Graca et al., 2018). On the other hand, however, gliosis after nervous impairment including spinal cord and retina was widely known as an obstacle for the regeneration of neuron and neural dendrites (Bringmann et al., 2006; Wu et al., 2011).

In the present study, apart from the retinal gliosis, there emerged a certain amount of GFAP-positive neural fibers within the SRS following the NSCs transplantation and the combined transplantation. This NSC-derived gliosis might impede the neural regeneration and the repairing effect resulting from transplanted stem cells. As NSCs were able to differentiate into glial cells, these neural fibers most likely came from the differentiation of NSCs. Basically, although NSCs were widely used to treat various neurodegenerative diseases, the maintaining of stemness had become an imposing barrier. To compensate the drawback, a promising way was to use another kind of stem cell to influence NSCs. Study had shown that coculture of human NSCs with human mesenchymal stem cells could significantly extend the stemness of NSCs via activating Notch-1 signal transduction (Haragopal et al., 2015). In the present study, with *in vitro* Transwell system, the stemness of NSCs was enhanced when OECs and NSCs were cocultured. Combined transplantation of OECs and NSCs also repressed the NSC-derived gliosis within SRS, illustrating that the differentiation of NSCs was inhibited and the stemness of NSCs was maintained by OECs. This gliosis regulating effect of OECs can be attributed to the main function of OECs as supporting cells. However, relatively low retina-protection effect was also observed following OEC single transplantation. As mentioned above, reactive gliosis can also bring about positive effect to retinal repair; inhibition of gliosis might be the cause of the low pro-retina activity. It should be noted that the underlying molecular mechanisms relating to endogenous stem cell activation as well as gliosis inhibition after cotransplantation of OECs and NSCs were not explored in the current study, which should be further explored in the future.

Besides, microglia show close relationship to the gliosis of Müller cells (Gao et al., 2015). As both Müller cells and microglia are responsible for the secretion of neurotrophic factor within the retina, the network formed by microglia–Müller glia–photoreceptors can significantly influence the microenvironment during retinal degeneration (Harada et al., 2002). On the one hand, degenerated photoreceptors induce the activation and migration of microglia from the inner to the outer retina. During this procedure, activated microglia alter the trophic factor production and further cause the gliosis of Müller glia, which can influence the production of neurotrophic factor in Müller glia (Harada et al., 2002). On the other hand, gliosis of Müller glia triggered by photoreceptor degeneration also leads to the apoptosis of neurons resulting in more severe activation of microglia (Telegina et al., 2018). These aspects trap the microglia–Müller glia–photoreceptor network into a vicious cycle and cause the deficiency of trophic factors, which is detrimental to the survival of photoreceptors. However, after combined transplantation of OECs and NSCs, gliosis was found to be reduced in the present study. Besides, our previous work confirmed the inhibition of microglia activation following NSCs transplantation (Li et al., 2016). These situations might improve the neurotrophic factor secretion situation and improve the photoreceptor survival subsequently.

The migration of transplanted cells is essential for the development of their function in the transplanted area. Migration exists in two dimensions: horizontal and vertical. Horizontal migration determines the scope of transplantation (McGill et al., 2012; Peng et al., 2014), while vertical migration determines the function of the inner retinal layer (Santos-Ferreira et al., 2016). Our previous results confirmed that better retinal preservation effects can be derived from combined transplantation due to the enhanced horizontal and vertical migration of transplanted cells (Qu et al., 2017). In the present study, differences in cell migration were also observed between the combined and the single transplantation groups. Grafted cells reached further following combined transplantation. Moreover, NSCs were able to migrate into the inner layer of the retina in the presence of OECs, although this was rarely observed when NSCs were cultured alone. A study showed that, during physiological development, the gene expression products of OECs, including Nelf and Semaphorin 4, are responsible for neuronal migration within the CNS of mice (Geller et al., 2013), indicating the potential migration-enhancing ability of OECs. In addition, OECs are fundamentally characterized by their ability to promote axonal regeneration (Yang et al., 2015). Our previous work also confirmed the stimulating effect on neuronal survival and the outgrowth of OECs, which was due to the phagocytosis of cell debris from OECs (Li et al., 2017). In this way, the processes and integration of NSCs might be extended and promoted following cotransplantation with OECs.

What is more, the fate of transplanted cells should also be taken into consideration. The main functions of NSCs transplanted into the subretinal space in RCS rats were phagocytosis of photoreceptor outer segments, secretion of neurotrophic factors, and inhibition of microglia (McGill et al., 2012; Jung et al., 2013; Li et al., 2016). Although NSCs were observed to be differentiated into photoreceptors and opsin-positive retinal cells, integration of NSC into ONL was hardly found (Nishida et al., 2000; Lin et al., 2014). As for OECs, our previous study showed that the function of OECs transplantation into the subretinal space of RCS rats mainly consisted of two aspects: (1) the microenvironment regulation effects, including secretion of neurotrophic factors and inhibition of the formation of reactive oxygen species; (2) the suppression of the gliotic injury response of the Müller cells (Huo et al., 2011, 2012; Xue et al., 2017). However, the differentiation and integration of OECs following retinal transplantation was not observed. Although there was better retina-protection effect after combined transplantation of OECs and NSCs, current study still presented a decreasing trend in both functional and morphological results. Considering the immune response following exotic cell transplantation, we speculate that the major ending of NSCs and OECs is the apoptosis after a certain time period. Moreover, we could not rule out the possibility that the greater improvements in the cotransplanted condition might be due simply to increased numbers of transplanted cells. The differences in responses to transplants of each cell type separately suggested that the greater improvements were more likely to be due to the combined effects of the two cell types.

Besides, in our previous study, transplantation of OECs mixed with ONFs had been performed. Results showed that OECs (but not ONFs) phagocytose porcine photoreceptor outer segments. The phagocytosis ability was even stronger than RPE (Huo et al., 2012). However, Li et al. also found that OECs and ONFs had synergistic effects in promoting axon regeneration (Li et al., 2005). In the present study, there is roughly 50/50 mixture of OECs and fibroblasts in the transplanted cells, which accords with the cell ratio in our previous study (Huo et al., 2011). OECs are known for their properties like interacting with the glial scar, stimulating angiogenesis, and promoting axonal outgrowth (Roet and Verhaagen, 2014; Gomez et al., 2018). Fibroblasts, both from the olfactory bulb and other parts of the body, can affect the function of other cells and present a lot of different effects. However, as the purpose of this study is to focus on the function of OECs, we referred the mixture of OECs/ONFs as OECs.

Since OEC and NSC transplantation have both entered into the clinical research stage, the advancement of the combined transplantation of OECs and NSCs from bench to bedside in the future is promising. As the main effect of cotransplantation was the activation of endogenous stem cells and improvements in the microenvironment, this therapeutic method could prospectively benefit all types of RDDs as well as other retinal lesions, including glaucoma and ocular trauma. Further research is required to confirm this possibility.

## Conclusion

In summary, the combined transplantation of NSCs and OECs better preserved retina than transplantation with NSCs or OECs alone, and this effect was verified by improved ERGs and increased ONL thickness in the combined transplantation group. Increased endogenous stem cell activation, better maintenance of NSC stemness, and enhanced migration of transplanted cells following combined transplantation might be potential mechanisms. All of these results illustrated that the combined transplantation of NSCs and OECs might be a possible alternative for the treatment of RDDs.

## Data Availability Statement

All the data used to support the findings of this study are available from the corresponding author upon reasonable request.

## Ethics Statement

The animal study was reviewed and approved by the Institutional Review Board of the Third Military Medical University.

## Author Contributions

HX and ZY contributed to the design of the project. WZ and LG contributed to the *in vivo* experiments and discussed the results. WZ, YL, QL, and YZ contributed to the *in vitro* experiments. LG and HX summarized the data and contributed in manuscript preparation. All authors read and approved the manuscript.

## Conflict of Interest

The authors declare that the research was conducted in the absence of any commercial or financial relationships that could be construed as a potential conflict of interest.
