# Supplementary material for: Combined Transplantation of Olfactory Ensheathing Cells With Rat Neural Stem Cells Enhanced the Therapeutic Effect in the Retina of RCS Rats
Source: Front Cell Neurosci. 2020 Mar 24;14:52. doi: 10.3389/fncel.2020.00052 (PMC7105604; doi:10.3389/fncel.2020.00052)
Supplement: Supplementary file 1 [file Data_Sheet_1.PDF]

Supplementary Material

Supplementary Figures

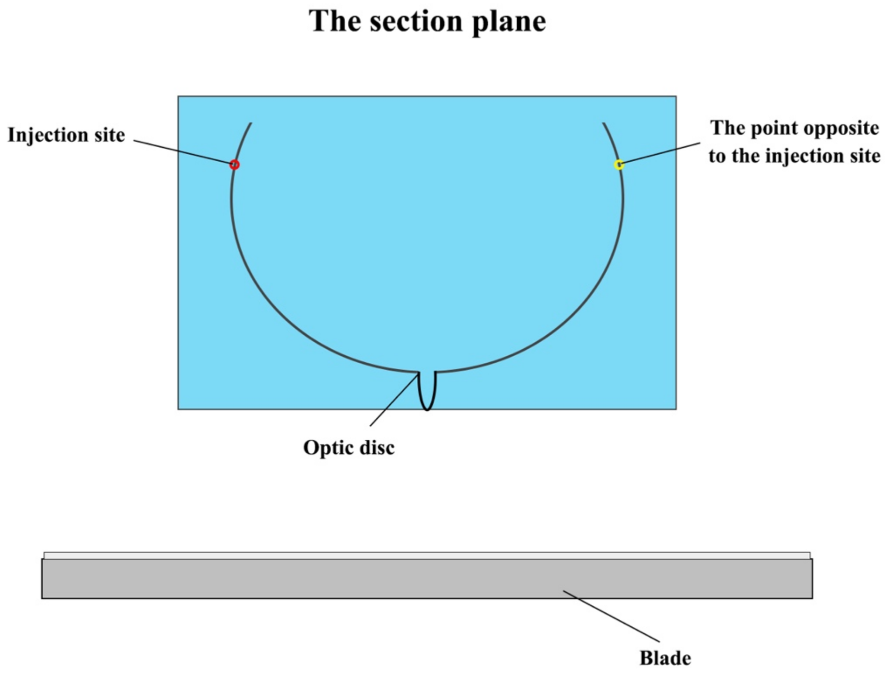

S Figure 1 The schematic diagram of the section plane of the reina.

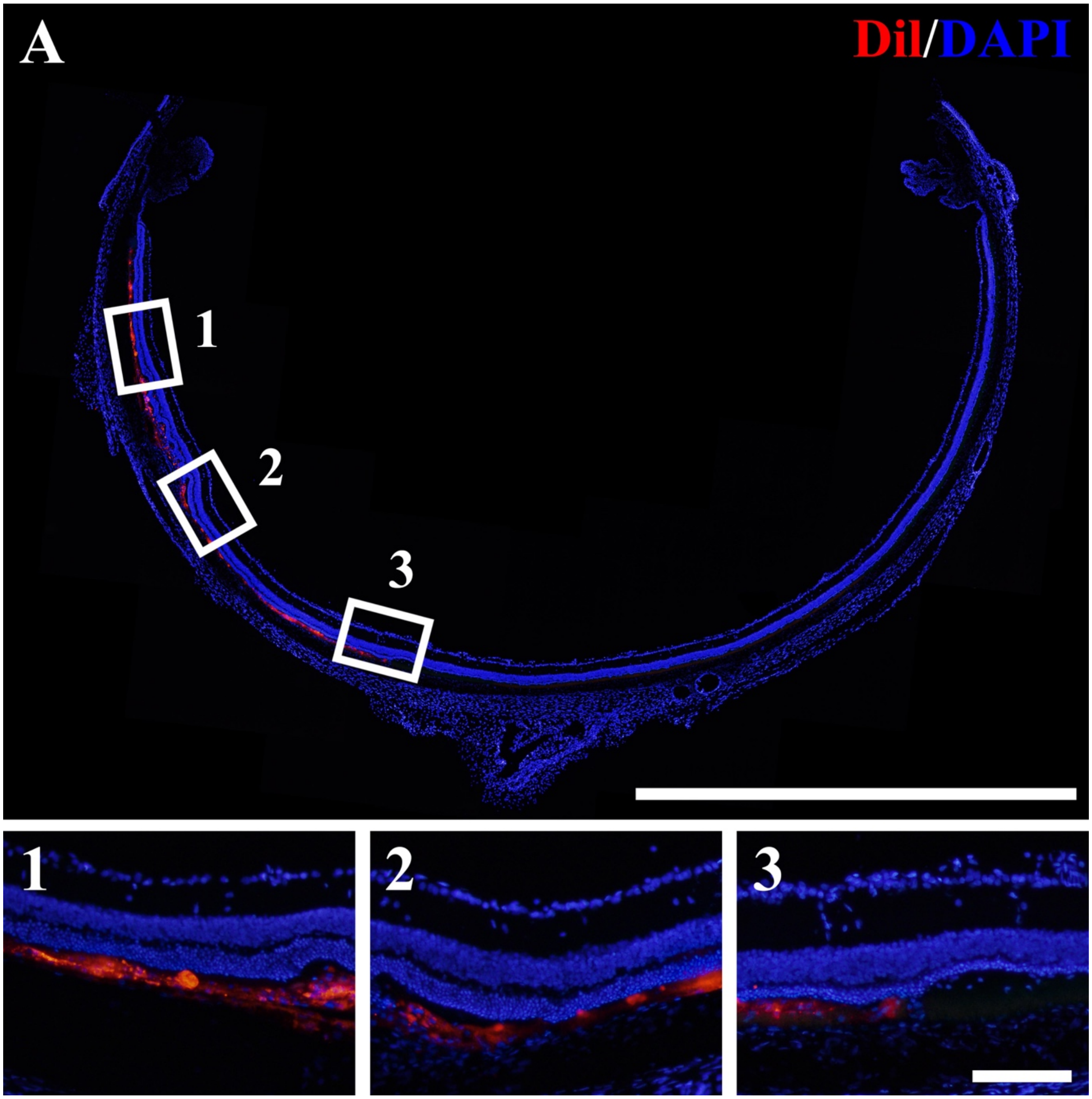

S Figure 2 The illustration of ONL thickness measurement. **A**: Whole retina sections. **1-3**: Three different nasal sides were chosen to calculate the ONL thickness. Scale bar: A: 2 mm; 1-3: 50 μm.

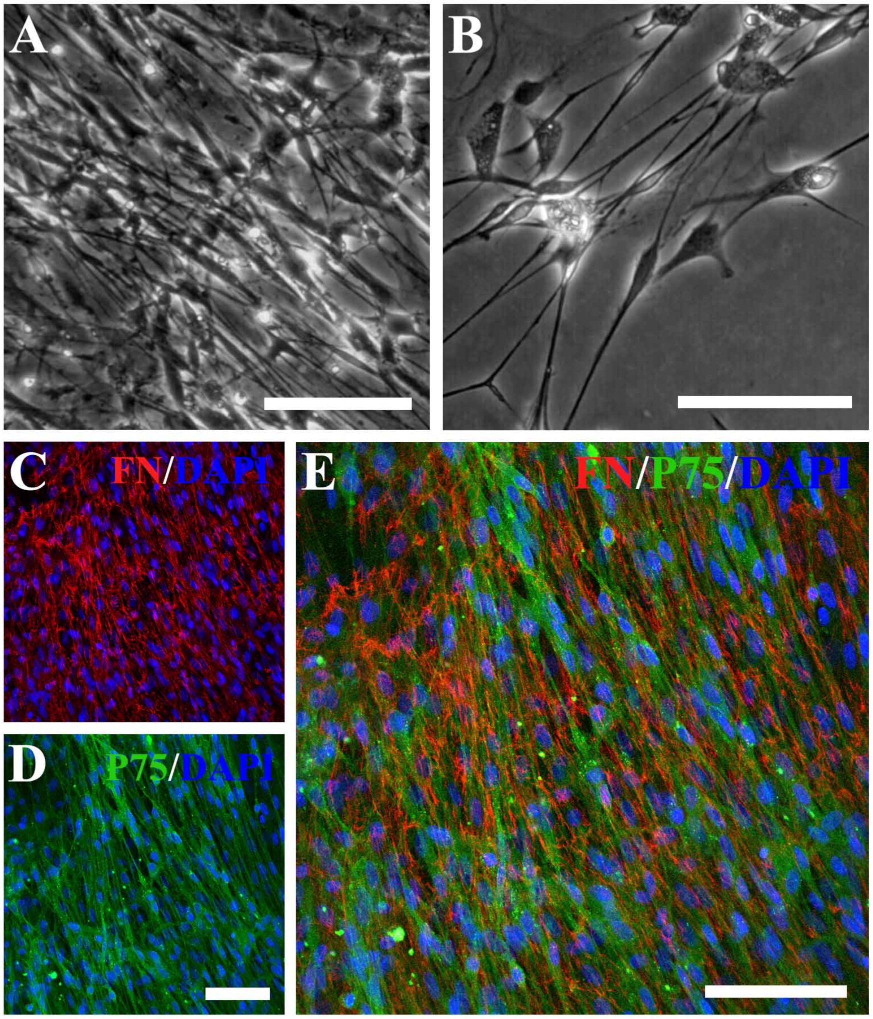

S Figure 3 Identification of rat OECs. **A-B**: Optical microscopy of OECs. **C**: Immunofluorescence staining of FN in isolated cells. **D**: Immunofluorescence staining of P75 in isolated cells. **E**: Merge of C and D. Scale bars: A: 40 μm; B: 10 μm; C-D: 25 μm. E: 25 μm.

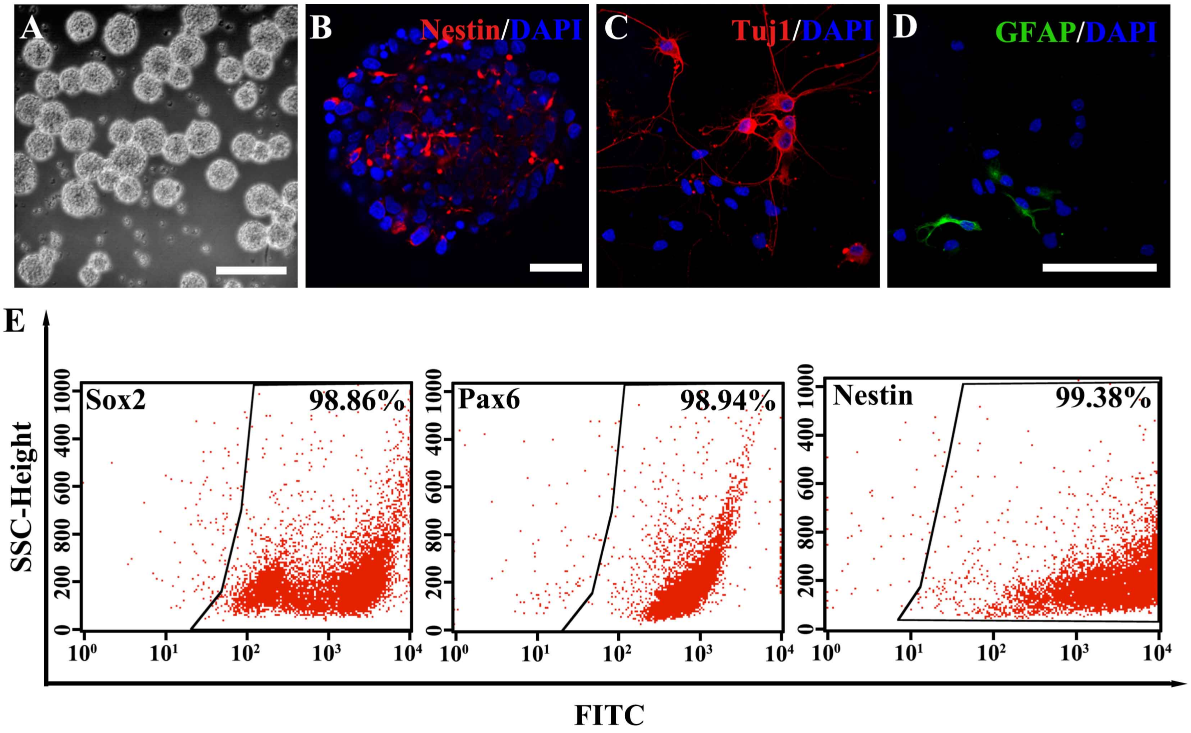

S Figure 4 Identification of rat NSCs. **A**: Optical microscopy of NSCs in sphere forms. **B**: Immunofluorescence staining of Nestin in NSCs. **C**: Immunofluorescence staining of Tuj1 in NSCs. **D**: Immunofluorescence staining of GFAP in NSCs. **E**: Flow cytometry analysis of NSCs with Sox2, Pax6, Nestin. Scale bars: A: 200 µm; B: 20 µm; C-D: 50 µm.
